# Comparative Genomic and Phylogenomic Analyses Reveal a Conserved Core Genome Shared by Estuarine and Oceanic Cyanopodoviruses

**DOI:** 10.1371/journal.pone.0142962

**Published:** 2015-11-16

**Authors:** Sijun Huang, Si Zhang, Nianzhi Jiao, Feng Chen

**Affiliations:** 1 CAS Key Laboratory of Tropical Marine Bio-resources and Ecology, RNAM Center for Marine Microbiology, South China Sea Institute of Oceanology, Chinese Academy of Sciences, Guangzhou, Guangdong, China; 2 State Key Laboratory of Marine Environmental Science, Xiamen University, Xiamen, Fujian, China; 3 Institute of Marine and Environmental Technology, University of Maryland Center for Environmental Science, Baltimore, Maryland, United States of America; ContraFect Corporation, UNITED STATES

## Abstract

Podoviruses are among the major viral groups that infect marine picocyanobacteria *Prochlorococcus* and *Synechococcus*. Here, we reported the genome sequences of five *Synechococcus* podoviruses isolated from the estuarine environment, and performed comparative genomic and phylogenomic analyses based on a total of 20 cyanopodovirus genomes. The genomes of all the known marine cyanopodoviruses are highly syntenic. A pan-genome of 349 clustered orthologous groups was determined, among which 15 were core genes. These core genes make up nearly half of each genome in length, reflecting the high level of genome conservation among this cyanophage type. The whole genome phylogenies based on concatenated core genes and gene content were highly consistent and confirmed the separation of two discrete marine cyanopodovirus clusters MPP-A and MPP-B. The genomes within cluster MPP-B grouped into subclusters mainly corresponding to *Prochlorococcus* or *Synechococcus* host types. Auxiliary metabolic genes tend to occur in a specific phylogenetic group of these cyanopodoviruses. All the MPP-B phages analyzed here encode the photosynthesis gene *psbA*, which are absent in all the MPP-A genomes thus far. Interestingly, all the MPP-B and two MPP-A *Synechococcus* podoviruses encode the thymidylate synthase gene *thyX*, while at the same genome locus all the MPP-B *Prochlorococcus* podoviruses encode the transaldolase gene *talC*. Both genes are hypothesized to have the potential to facilitate the biosynthesis of deoxynucleotide for phage replication. Inheritance of specific functional genes could be important to the evolution and ecological fitness of certain cyanophage genotypes. Our analyses demonstrate that cyanopodoviruses of estuarine and oceanic origins share a conserved core genome and suggest that accessory genes may be related to environmental adaptation.

## Introduction

Viruses are the most abundant biological entities in the ocean, and could affect the population structure and evolution of their hosts [1–3]. Cyanophage are a group of viruses that infect cyanobacteria. They have been recognized as an important biological factor that influences the abundance, diversity and productivity of picocyanobacteria *Synechococcus* and *Prochlorococcus* in the ocean [4–7]. In the past two decades, many cyanophages that infect marine *Synechococcus* and *Prochlorococcus* have been isolated and characterized, and known marine cyanophages are tailed double-stranded DNA viruses, belonging to three well-defined bacteriophage families: *Myoviridae*, *Podoviridae* and *Siphoviridae* [6, 8–16].

Cyanopodoviruses are highly host specific and have been extensively found in various marine habitats [8, 12–14], representing a ubiquitous and ecologically important viral fraction in the ocean. To date, a total of 12 complete cyanopodovirus genomes have been reported [17–20]. According to comparisons based on gene content and genome architecture, all known marine cyanopodoviruses are similar to archetypical coliphage T7, thus denoted as T7-like (the viral genus “T7-like viruses” has been renamed to “*T7likevirus*” from 2012 by International Committee on Taxonomy of Viruses) cyanophages. A few cyanopodovirus-encoded genes were found to be related to metabolic processes of hosts, such as photosynthesis, pentose phosphate pathway, phosphorus acquisition and carbon metabolism [19–22]. Recently, these phage-encoded host-like genes were delineated as auxiliary metabolic genes (AMGs) [23]. One of the AMGs, *psbA*, was shown to be expressed during infection and is thought to be able to confer fitness benefits to cyanophages [24–26].

The DNA polymerase gene (DNA *pol*) was used to investigate the genetic diversity of marine cyanopodoviruses and two marine picocyanobacterial podovirus clusters (MPP-A and MPP-B) were established [13]. This classification was also supported by a recent phylogenomic analysis mainly based on *Prochlorococcus* podoviruses [19]. Using this molecular marker, genetic diversity and temporal and spatial variations of marine cyanopodovirus community were described [14, 27, 28].

Despite the fact that a number of cyanopodovirus genomes have been delineated, however, within them, much fewer genomes were from *Synechococcus* podoviruses (n = 2) than from *Prochlorococcus* podoviruses (n = 10). Especially, only one genome of cyanopodovirus (*Synechococcus* phage P60) isolated from estuarine environment was described. Estuarine ecosystems such as the Chesapeake Bay harbor picocyanobacterial communities which are distinct from those in the open oceans [29, 30]. Although the MPP-B cluster contains the most numerically dominant cyanopodoviruses in the sea [14, 27, 28], no *Synechococcus* podovirus genome in this cluster has been described thus far. Currently, the MPP-A cluster only contains three cyanopodoviruses with known genomes. Therefore, additional genome sequences from *Synechococcus* podoviruses will deepen our understanding on the evolution of picocyanobacterial podoviruses and the relationship between genomes from MPP-A and MPP-B clusters.

In this study, we described four complete genome sequences of podoviruses that were isolated from the Chesapeake Bay using the estuarine *Synechococcus* strains, and one genome of podovirus infecting oceanic *Synechococcus*. Comparative genomic and phylogenomic analyses were performed based on the 20 known cyanopodovirus genomes including 9 *Synechococcus* podoviruses and 11 *Prochlorococcus* podoviruses. We classified the core- and pan-genomes and assessed the phylogenomic relationships among these genomes. Gene content variation among different clusters or subclusters was demonstrated and discussed.

## Materials and Methods

### Phage isolation and DNA extraction and sequencing

Five cyanopodoviruses (S-CBP1, S-CBP2, S-CBP3, S-CBP4 and S-CBP42) isolated from the Chesapeake Bay estuary [13, 27] were selected for genome sequencing. Phage propagation, harvesting and DNA preparation followed the methods described by Wang and Chen [13]. Genomes of S-CBP2, S-CBP3, S-CBP4 and S-CBP42 were sequenced and assembled using the 454 pyrosequencing platform at the Broad Institute [31]. Genome of S-CBP1 was sequenced at Majorbio Biotech (Shanghai, China) using ABI 3730XL DNA Analyzer and assembled using the Phred/Phrap package (http://www.phrap.org).

### Comparative genomics

Programs GeneMark [32] and Glimmer [33] were used to predict the open reading frames (ORFs). Protein sequences of ORFs were input to perform BLASTP comparisons against the NCBI nr protein database and potential functions were then assigned based on best hits. We performed an “all-to-all” BLASTP (-p blastp -W 3 -a 8 -e 0.001 -G 11 -E 1 -F F -U F -M BLOSUM62) comparison of the 20 cyanopodovirus proteomes (Table 1). Orthologous relationship of any pairwise sequences was assigned when their reciprocal BLASTP hits met the cutoff e-value ≤ 1e-5 and alignment length covered at least 50% of the shorter sequence. For short sequences less than 100 amino acids, orthologous relationship was also determined when BLAST identity was ≥ 35% even if the e-value was not ≤ 1e-5. HMM profiles [34] were built for highly divergent genes (e.g. genes coding for a putative tail fiber and internal capsid proteins) by using HMMBUILD, and the resulting protein databases were searched by using HMMSEARCH and significant similarity was determined when *E*-value was ≤ 1e-5. A core gene represents an clustered orthologous group (COG) that is shared by all the 20 cyanopodoviruses. A pan-genome represents all the COGs (including singletons) found in a specific number of genomes. Pan- and core-genomes were plotted as a function of the number of genomes analyzed by using R scripts. Genome maps were created based on the outputs of genome annotations using Canvas v12. T-test was performed by using the SPSS software.

**Table 1:** Summary of marine cyanopodovirus genomes.

Group	Phage	Accession #	Original host	Host clade	Genome size (bp)	# ORFs	%G+C	Site of origin	Collection date	Depth (m)	Publication
	P-RSP2	HQ332139	*Prochlorococcus* MIT9302	HLII	42257	48	34.0%	Red Sea	14-Jul-96	surface	[19]
MPP-A	P60	AF338467	*Synechococcus* WH7805	5.1-VI	46675	55	53.3%	Satilla River Estuary	16-Jul-88	surface	[17]
	Syn5	EF372997	*Synechococcus* WH8019	5.1-II	46214	61	55.0%	Sargasso Sea	30-Nov-86	surface	[18]
	P-SSP9	HQ316584	*Prochlorococcus* SS120	LLII	46997	54	40.5%	BATS	31-Aug-95	100	[19]
	S-CBP2	KC310806	*Synechococcus* CB0208	5.2-CB4	46237	53	55.0%	Chesapeake Bay	27-Sep-02	surface	This study
	S-CBP42	KC310805	*Synechococcus* WH7803	5.1-V	45218	57	54.6%	Chesapeake Bay	05-Jun-06	surface	This study
MPP-B	P-SSP7	AY939843	*Prochlorococcus* MED4	HLI	44970	54	38.8%	Sargasso Sea	01-Sep-99	100	[20]
	P-SSP5	GU071100	*Prochlorococcus* MIT9515	HLI	47055	55	39.2%	North Pacific gyre	Sep-99	120	unpublished
	P-RSP5	GU071102	*Prochlorococcus* NATL1A	LLI	47741	68	38.7%	Red Sea	13-Sep-00	130	[19]
	P-HP1	GU071104	*Prochlorococcus* NATL2A	LLI	47536	66	39.9%	HOT	8-Mar-06	25	[19]
	P-SSP2	GU071107	*Prochlorococcus* MIT9312	HLII	45890	59	37.9%	BATS	31-Aug-95	120	[19]
	P-GSP1	HQ332140	*Prochlorococcus* MED4	HLI	44945	53	39.6%	Gulf Stream	Aug-95	80	[19]
	P-SSP3	HQ332137	*Prochlorococcus* MIT9312	HLII	46198	56	37.9%	BATS	31-Aug-95	100	[19]
	P-SSP10	HQ337022	*Prochlorococcus* NATL2A	LLI	47325	52	39.2%	BATS	5-Jun-96	100	[19]
	P-SSP11	HQ634152	*Prochlorococcus* MIT9515	HLI	47039	54	39.2%	BATS	1-Sep-99	100	[19]
	S-CBP1	KC310802	*Synechococcus* CB0101	5.2-CB4	46547	51	47.6%	Baltimore Inner Harbor	16-Jul-02	surface	This study
	S-CBP3	KC310803	*Synechococcus* CB0101	5.2-CB4	45871	55	47.0%	Chesapeake Bay	12-Jul-04	surface	This study
	S-CBP4	KC310804	*Synechococcus* CB0101	5.2-CB4	44147	49	44.4%	Chesapeake Bay	15-Jul-07	surface	This study
	S-RIP1	HQ317388	*Synechococcus* WH8101	5.1-VIII	44892	54	42.9%	Narragansett Bay	26-Sep-07	surface	unpublished
	S-RIP2	HQ317389	*Synechococcus* WH7803	5.1-V	45728	56	47.3%	Rhode Island Sound	22-Oct-07	surface	unpublished

### Whole genome tree and tree comparison

Four methods were implemented to infer phage whole genome trees. *i)* A phylogenetic tree based on the concatenated core genes was built by PAUP* using the distance criterion. A heuristic search with 1000 bootstrap replications was conducted in this analysis. *ii)* The maximum likelihood (ML) trees for each of the core genes were also constructed by RAxML [35, 36] using the JTT protein substitution matrix and the GTRGAMMA+I model to estimate the proportion of invariable sites and the resulting trees were subsequently loaded to the CONSENSE program in PHYLIP package [37] to infer a consensus tree using the extended majority rule. *iii)* A dendrogram was built by SplitsTree4 [38] using ML distance measurement based on gene content. *iv)* Whole genome network was constructed with a ML distance estimator and represented as a neighbor net as implemented by SplitsTree4. For the methods *i)* and *ii)*, Clustal X2 [39] was used to align the sequences and the resulting alignments were trimmed to remove highly divergent regions by the program Gblocks [40]. The topological distances among phylogenetic trees for core genes were calculated based on the symmetric difference as implemented in TREEDIST in PHYLIP. The resulting distance matrix was loaded to PRIMER5 (http://www.primer-e.com/) to assess similarity relationships among phylogenetic trees using non-metric multidimensional scaling (NMDS).

### Phylogenies of the thymidylate synthase gene

Sequences of the thymidylate synthase gene *thyX* were retrieved from cyanobacterial and cyanophage genomes. The protein sequences were aligned using Clustal X2 [39] and ML trees were then built using MEGA6 [41] with the model JTT+GAMMA+I. Bootstrap tests were performed for 100 replicates.

### Nucleotide sequence accession number

The complete genome sequences of cyanopodoviruses S-CBP1, S-CBP2, S-CBP3, S-CBP4 and S-CBP42 have been deposited in the GenBank database under accession numbers KC310802, KC310806, KC310803, KC310804, and KC310805.

## Results and Discussion

### General features of cyanopodovirus genomes

Complete genome sequences of four *Synechococcus* podoviruses (S-CBP1, S-CBP2, S-CBP3, and S-CBP4) which infect Chesapeake Bay *Synechococcus* strains were obtained. S-CBP1, S-CBP3 and S-CBP4 were isolated from the Chesapeake Bay on *Synechococcus* strain CB0101, while S-CBP2 was isolated from the Bay on *Synechococcus* strain CB0208 [13, 27]. In addition, we also sequenced the genome of S-CBP42, a podovirus which infects oceanic strain *Synechococcus* WH7803 [27] (Table 1). Previously, 12 complete genomes of marine cyanopodoviruses were reported [17–19], and three other genomes have been released in the GenBank (Table 1). Thus, among the 20 cyanopodoviruses with known genome, six (the five described above and *Synechococcus* podovirus P60) were isolated from estuarine waters and the others were from oceanic waters.

**Fig 1:**
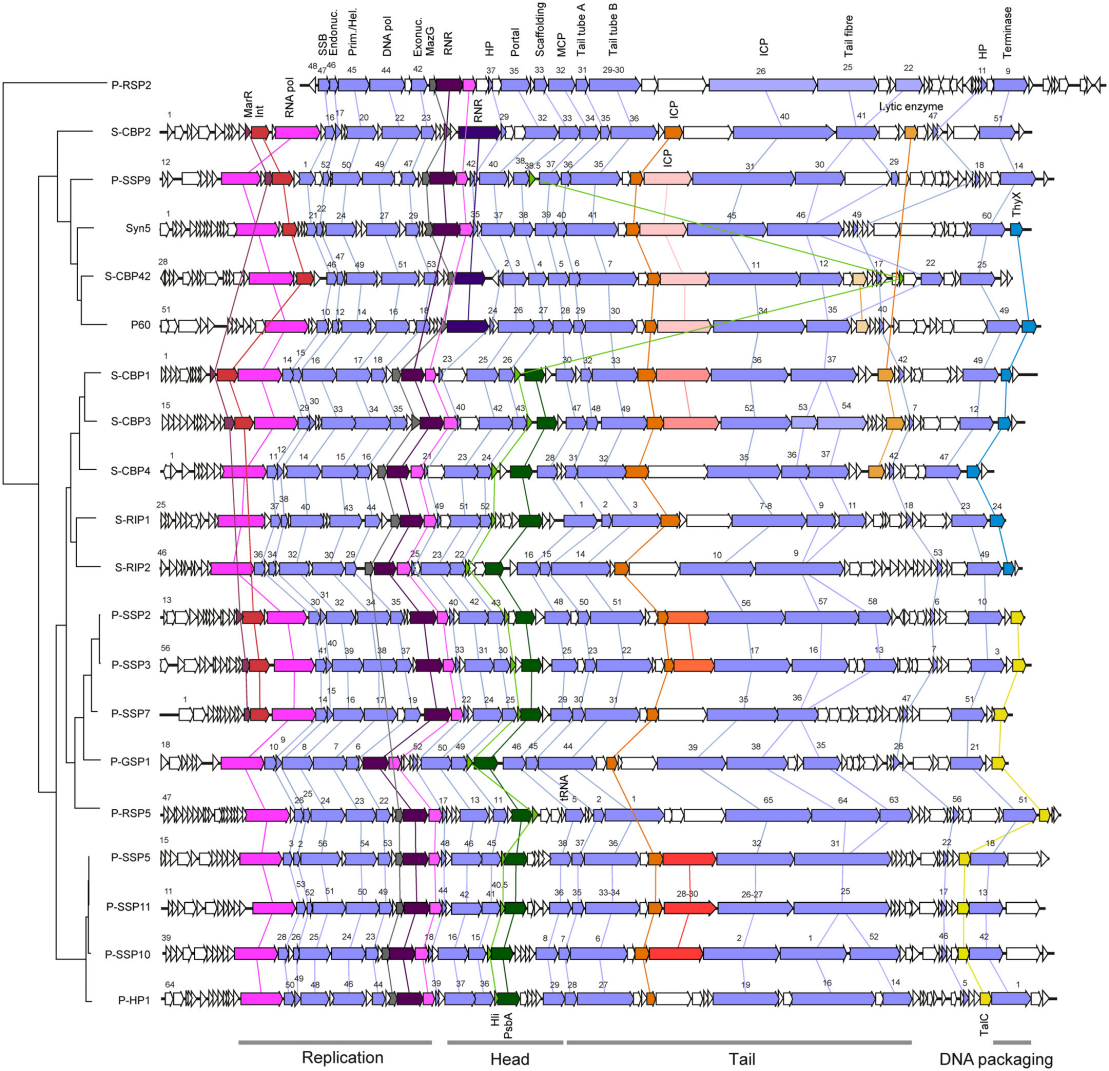
Alignment of the 20 marine cyanopodovirus genomes. Core genes are indicated by light blue arrows. The other arrows that are colored and linked by lines represent a few shared non-core genes with known or putative function. Abbreviation: MarR, MarR family transcriptional regulator; RNA pol, RNA polymerase; SSB, single-stranded DNA binding protein; endonuc., endonuclease; prim./hel., primase/helicase; DNA pol, DNA polymerase; exonuc., exonuclease; MazG, pyrophosphatase; RNR, ribonucleotide reductase; Hli, high light inducible protein; PsbA, photosystem II D1 protein; MCP, major capsid protein; ICP, internal core protein; TalC, transaldolase; ThyX, thymidylate synthase; HP, hypothetical protein.

In general, marine cyanopodoviruses have a conserved genome size ranging from 42.3 to 47.7 kilo base pair (kbp), which is larger than the size of typical T7-like phages infecting heterotrophic bacteria (37.4 to 39.9 kbp) (data from NCBI GenBank) and freshwater cyanopodoviruses (40.9 to 43.2 kbp) [42, 43]. The *Prochlorococcus* podoviruses have a significant lower G+C content (34–40.5%, Mean = 38.6%, Standard Deviation (SD) = 1.7%, N = 11) than marine *Synechococcus* podoviruses (43–55%, Mean = 49.7%, SD = 4.8%, N = 9) (Table 1) (T-test, *P* < 0.01), reflecting the lower G+C content of *Prochlorococcus* than marine *Synechococcus* [44, 45]. Such a G+C distribution pattern suggests that podoviruses infecting marine *Synechococcus* and *Prochlorococcus* may follow different virus-host co-evolution paths. Generally, the genome sequences of these cyanopodoviruses are highly syntenic (Fig 1, homologous genes were connected by colored lines between genomes), suggesting that those genomes have very similar architectures. The homogeneity in genome organization and the high proportion of core genes (28% by gene number, 50% by genome size, see below) may reflect a constraint which could be an important force for marine cyanopodoviruses to maintain co-evolution with hosts.

### Pan- and core-genomes

**Fig 2:**
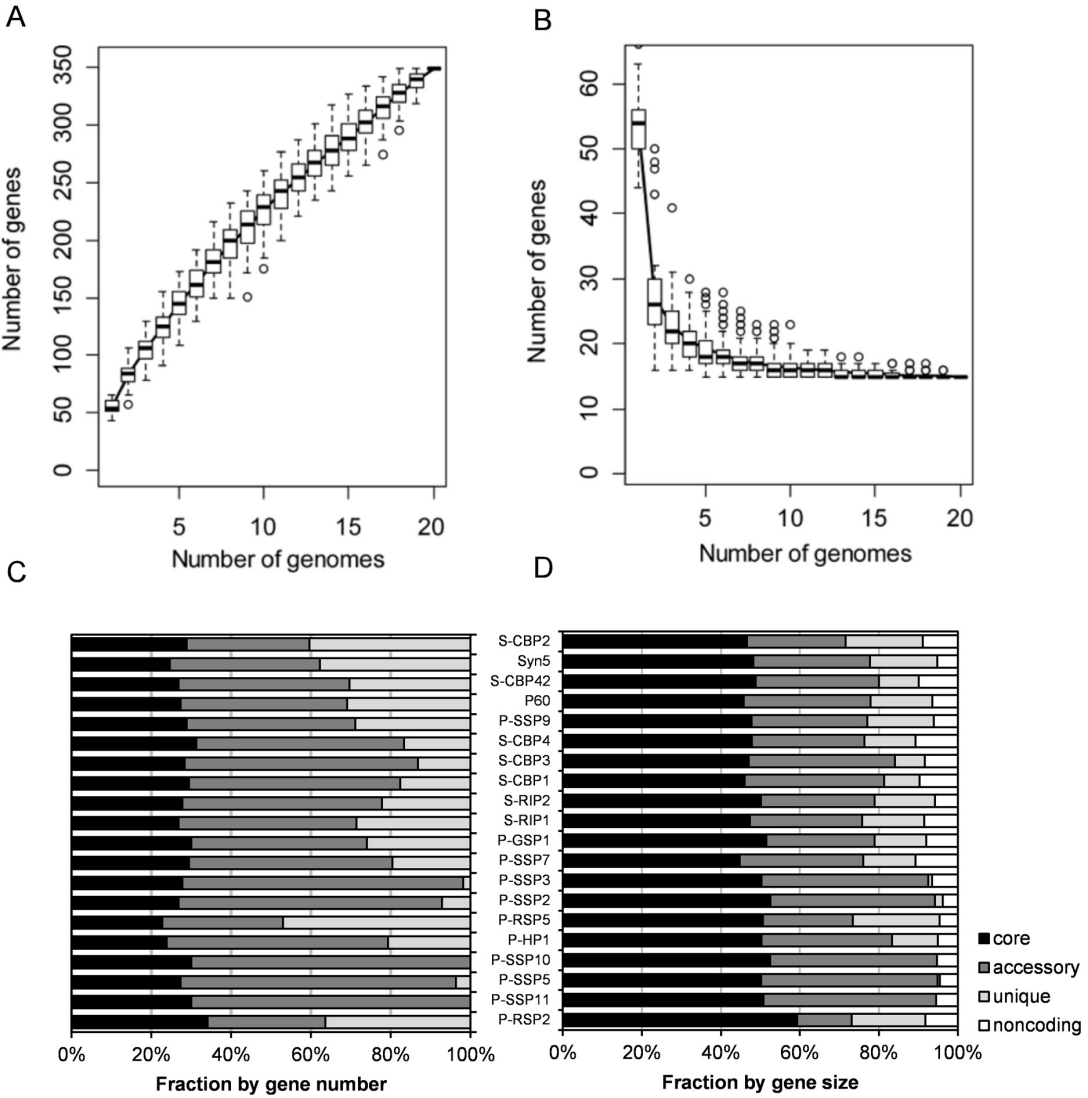
A and B. Pan- and core-genomes of cyanopodoviruses. The pan- (A) and core-genomes (B) were plotted as a function of the number of genomes analyzed. The pan-genome is the total number of genes of genomes in a subset sampled, while the core-genome is the genes shared by all genomes in the same subset. The line represents the average and the white box combing with dash lines represents estimated confidence interval. C and D. Fractions of core, accessory and unique genes of each genome.

A pan-genome of 349 COGs across all the 20 genomes was identified (Fig 2A, S1 File). This added additional 64 COGs into 285 COGs in the pan-genome of 12 marine cyanopodoviruses reported by Labrie and colleagues [19]. The gene accumulation curve was still far from being saturated (Fig 2A), suggesting the existence of vast unexplored genetic diversity of marine cyanopodoviruses. Similarly, the number of genes in the pan-genome of 28 cyanomyoviruses of the *T4likevirus* genus [46] and 12 *Prochlorococcus* [44] also appeared far from reaching a plateau. In contrast, the pan-genome size of *Streptococcus* was saturated for 26 genomes [47]. Pan-genome size depends on the level of genome sequence conservation and the number of genomes sampled. A larger number of cyanopodovirus genomes should be supplemented to estimate the pan-genome size of marine cyanopodoviruses.

Among the total 349 COGs, 15 were core genes that are shared by all the 20 cyanopodovirus genomes. These core genes are involved in virion structure and DNA replication and display remarkable synteny across the 20 genomes (Fig 1). Although an additional seven *Synechococcus* podoviruses were added into the analysis, the number of core genes has not decreased compared to the previous result [19]. It was also shown that the cumulative curve of core genes leveled off when 10 genomes were sampled (Fig 2B). Together, these results indicate that podoviruses infecting marine *Synechococcus* and *Prochlorococcus* share common conserved core genes, so do cyanopodoviruses isolated from brackish and oceanic waters. Our analysis suggests that the core gene set of marine cyanopodoviruses was well determined by known genomes.

**Fig 3:**
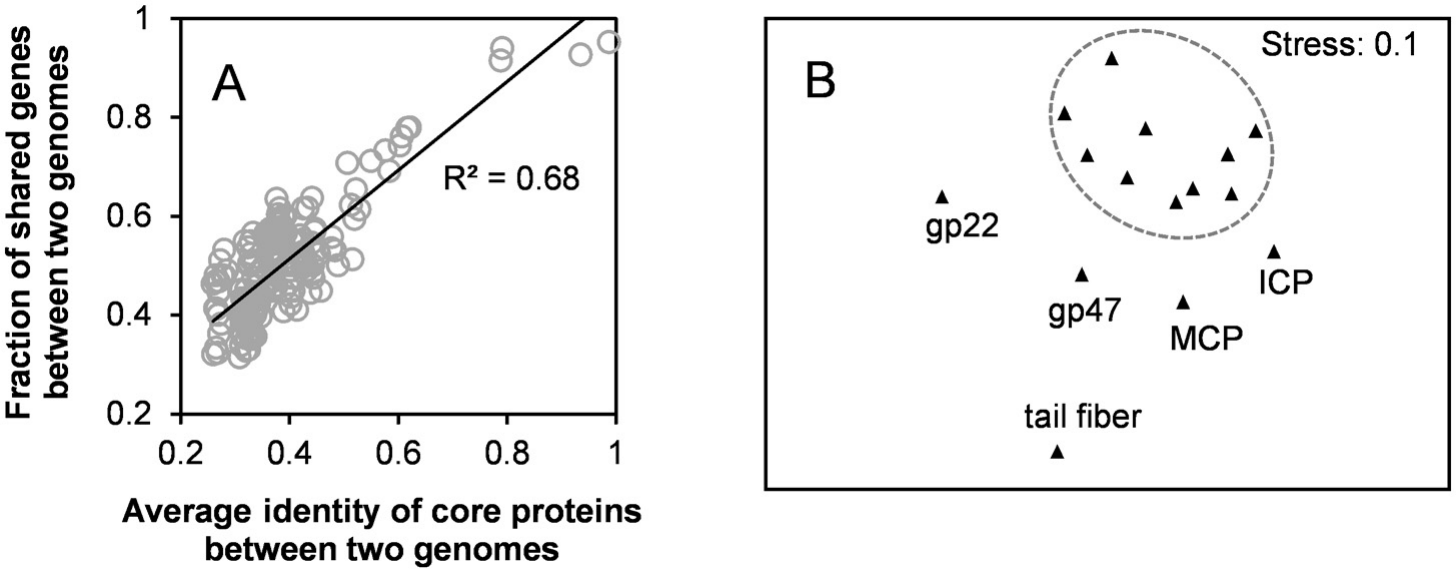
A. Linear relationship between the average protein sequence identity of core genes and the fraction of shared genes between two genomes. B. Multidimensional scaling showed the topological distances among the phylogenetic trees for core genes. The dash circle surrounds a relative more conserved core. Abbreviations refer to the legend of Fig 1.

Beside the 15 core genes, there were 99 accessory genes (shared by 2–14 genomes), and 235 unique genes (unique to a particular genome). On average, core, accessory and unique genes represented 28, 50, and 22% of total genes in each genome, respectively (Fig 2C). Due to relatively larger gene size of the core genes, they nearly made up 50% of each genome size (Fig 2D). Similarly, core genes make up 57% and 60% of the average genome sizes of marine *Synechococcus* [45] and *Prochlorococcus* [44], respectively. In contrast, core genes only account for 26% of the size of each cyanomyovirus genome [48, 49], on average, while marine cyanosiphoviruses comprise at least three distinct subtypes which do not share any core genes [50, 51]. The fraction of shared genes between two genomes showed a significant linear correlation to the average protein sequence identity of core genes between these two genomes (Fig 3A). Such a correlation indicates that the rate of gene gain and loss is positively correlated to the evolution rate of broadly shared genes, and further suggests that the fraction of core genes in a genome reflects the level of genome conservation. Together, our results suggest that known cyanopodovirus genomes are highly conserved among the three cyanophage types, with respect to the core genome proportion.

Interestingly, genes coding for a tail fiber protein, an internal core protein (ICP), the major capsid protein (MCP) and two hypothetical proteins (represented by gp22 and gp47 in P-SSP7) exhibited phylogenetic incongruence from the other 10 core genes (Fig 3B). It is possible that these five core genes are prone to more frequent genetic exchanges than the other 10 core genes. The genetic change on tail fiber gene may allow phages to adapt to rapidly changing host receptors [19]. In contrast, the *mcp* gene was thought to be among a more conserved gene regime, such as those of myoviruses [52] and cyanomyoviruses [53] of the *T4likevirus* genus. However, it is not clear why the *mcp* genes in cyanopodovirus are less conserved. The *mcp* genes of a few marine viruses have been used as molecular markers to explore the genetic diversity of specific viral groups, such as those of myoviruses [52] and cyanomyoviruses [54] of the *T4likevirus* genus. However, we suggest that the *mcp* gene of marine cyanopodovirus lacks enough conservation to serve as a molecular marker for diversity analysis.

### Whole genome phylogeny

**Fig 4:**
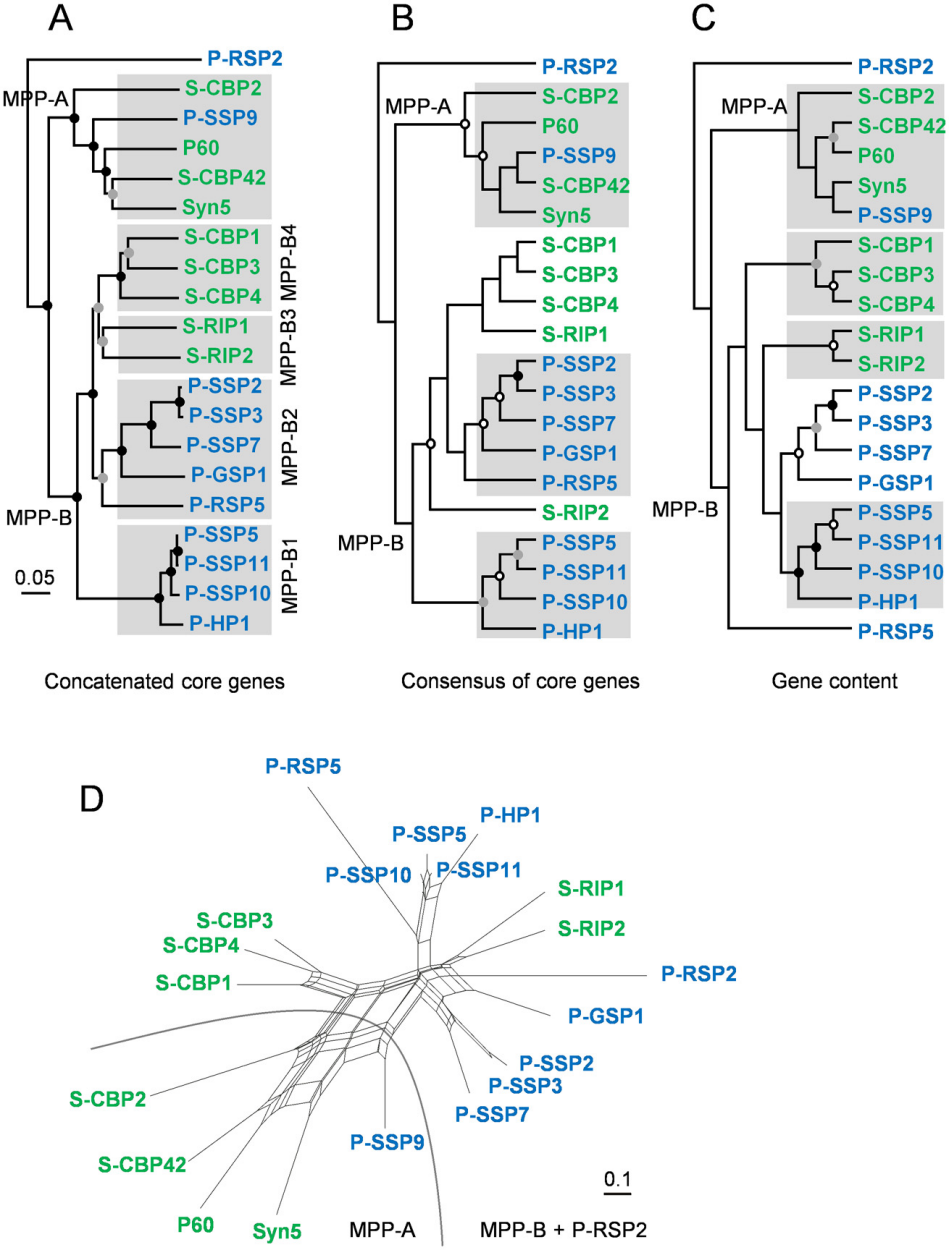
Whole genome phylogenies and network of cyanopodoviruses. A, a phylogenetic tree based on the concatenated core genes built by using the distance method; B, a consensus tree inferred from ML trees built for the 15 core genes; C, a dendrogram built by using ML distance measurement based on gene content; D, a whole genome network constructed based on gene content. *Synechococcus* podoviruses were shown in blue and *Prochlorococcus* podoviruses shown in green. Black, grey and open circles respectively represent bootstrap supports of 100%, 75–99% and 50–74%. The grey shading in panel A indicates cluster MPP-A and subclusters MPP-B1, B2, B3 and B4, and those cluster/subclusters that exist in panel B and C are also marked with shading.

We constructed phylogenies based on core gene alignments of cyanopodoviruses using three approaches (Fig 4A–4C) (see Materials and methods). Overall, significant congruence were observed among the tree constructed based on concatenated sequences of core genes (Fig 4A), the consensus tree of all core gene trees (Fig 4B) and the dendrogram based on gene content (Fig 4C). All these phylogenetic trees divided the 19 of the 20 cyanopodoviruses into two clusters, MPP-A and MPP-B, with the *Prochlorococcus* podovirus P-RSP2 as an outlier. This division agrees with the previous phylogenies built via a single DNA *pol* gene [13, 14] or the concatenated core genes of 12 genomes [19]. Most of MPP-A phages were isolated from *Synechococcus* while MPP-B phages from either *Synechococcus* or *Prochlorococcus* (Fig 4A–4C), in agreement with an observation based on more phage isolates [14].

In cluster MPP-B, *Prochlorococcus* and *Synechococcus* podoviruses were generally separated (Fig 4A–4C). The concatenated core gene phylogenies built by the distance method (Fig 4A) and the maximum likelihood method [55] are highly consistent, and both divided phages into four well supported subclusters (Fig 4A), two of which comprising *Prochlorococcus* podoviruses are identical to the subclusters (MPP-B1 and B2) defined previously [19]. The five *Synechococcus* podoviruses formed two independent subclusters (MPP-B3 and MPP-B4) in the MPP-B cluster (Fig 4A). Subcluster MPP-B3 consisted of three *Synechococcus* podoviruses (S-CBP1, S-CBP3 and S-CBP4) isolated from estuarine waters of the Chesapeake Bay and subcluster MPP-B4 contained two strains isolated from coastal waters (S-RIP1 and S-RIP2) (Fig 4A). The formation of four subclusters is also supported by the gene content dendrogram (Fig 4C). However, the consensus tree of core genes (Fig 4B) shows different clustering within the MPP-B cluster (Fig 4A and 4C). This is not surprising because at least five out of the 15 core genes have diverged evolutionary trajectories (Fig 3B).

The separation of clusters MPP-A and MPP-B and the divergence of four subclusters within cluster MPP-B were well supported by phylogenies based on core genes and based on gene content. It appears that the gene content variation resulted from gene gain and loss is significantly constrained by the phylogenetic relationship. This inference is in keeping with the result shown in Fig 3A. Such a pattern suggests that the horizontal gene transfer between the two cyanopodovirus clusters or among those subclusters is limited.

**Fig 5:**
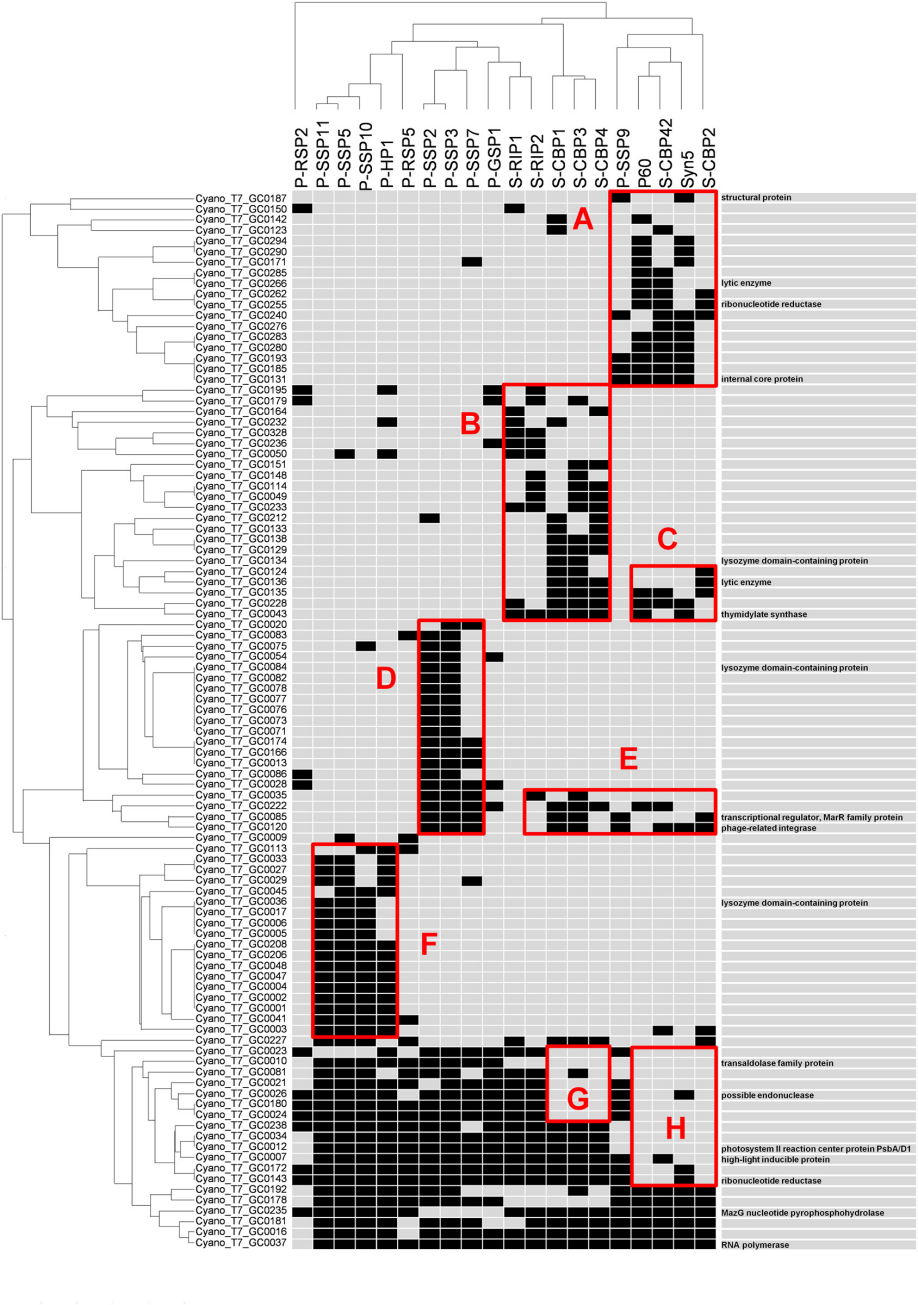
Distribution pattern of accessory genes (n = 99) among the 20 marine cyanopodovirus genomes. A black box represents a presence. Cyano_T7_GC stands for T7-like cyanophage gene cluster. The dendrograms were created based on the presence/absence matrix of accessory genes. The UPGMA and WPGMA methods were used to cluster the genes and the phages, respectively. The right column lists those genes with known/putative functions. Red boxes (A-H) indicate genes which were enriched or absent in certain phage groups. All the 349 COGs found among the 20 genomes were listed in S1 File.

The relationship among phage isolates in the phylogenetic network constructed based on gene content is similar to those observed in Fig 4A and 4C, with notable exception of the positions of S-RIP1, S-RIP2 and P-RSP2, which are grouped more closely with MPP-B *Prochlorococcus* podoviruses (Fig 4D). Interestingly, in this network, phages S-CBP1, S-CBP3, S-CPB4 and P-SSP9 appear to occupy the intermediate positions connecting MPP-A and MPP-B clusters (Fig 4D). This pattern is corresponding to the observation that certain similarities in presence/absence of accessory genes existed between MPP-B4 *Synechococcus* phages and MPP-A *Synechococcus* phages, as well as between *Prochlorococcus* phage P-SSP9 and MPP-B *Prochlorococcus* phages (Fig 5G and 5H). Despite falling within MPP-A cluster, P-SSP9 still has host-like G+C content that differs greatly from other *Synechococcus* MPP-A phages. In addition, it is noticeable that MPP-B4 phages and three out of five MPP-A phages (S-CBP2, S-CBP42 and P60) were isolated from estuarine waters, while S-RIP1 and S-RIP2 were from coastal waters. It is plausible that such network pattern may be in part related to host population or to the origin of isolating environment.

### Accessory genes

No obvious diagnostic features on the content of accessory genes could be found to distinguish *Prochlorococcus* and *Synechococcus* podoviruses, similar to marine cyanomyoviruses [48] (Fig 5). Moreover, no COGs exclusively obtained by MPP-A phages were observed and only two such COGs (*psbA* and a gene without a known function) existed in these MPP-B phages (Fig 5). Despite this, blocks of genes were indeed enriched (Fig 5A–5F) or lost (Fig 5G and 5H) in some specific phage groups.

Phage AMGs such as those coding for photosystem II D1 protein (PsbA), high light inducible protein (Hli), pyrophosphatase (MazG) and transaldolase (TalC) were found in the accessory gene fraction (Figs 1 and 5). In contrast, among marine cyanomyoviruses, *psbA* and *hli* are within the core set [48, 56]. Certain AMGs likely appear in specific phylogenetic groups. The most striking example is that *psbA* was present in all the MPP-B phages but absent in all the MPP-A phages and the outlier P-RSP2 (Figs 1 and 5). Dekel-Bird and colleagues [14] also reported that all known MPP-A isolates do not encode *psbA* while nearly all MPP-B phages contain *psbA*. *hli* was not only present in all MPP-B phages but also in two MPP-A phages, P-SSP9 and S-CBP42 (Figs 1 and 5). Different from other AMGs that are highly syntenic, *hli* in S-CBP42 is located ~20 kbp downstream from the locus of other *hli* genes (Fig 1). It appears that S-CBP42 lost the *hli* at the common *hli* locus but acquired another one at a downstream site. This agrees with the inference that *hli* could be transferred to phages multiple times [21]. *talC* was only present in *Prochlorococcus* podoviruses in the MPP-B cluster but none of *Synechococcus* podoviruses contained *talC* (Figs 1 and 5). *mazG* was present in all the cyanopodoviruses except the group comprising P-SSP2, P-SSP3, P-SSP7 and P-GSP1 (Figs 1 and 5). This distribution pattern of AMGs in cyanopodoviruses suggests that their acquisitions or losses in specific groups likely occurred around or after the time of divergence of MPP-A and MPP-B clusters.

**Fig 6:**
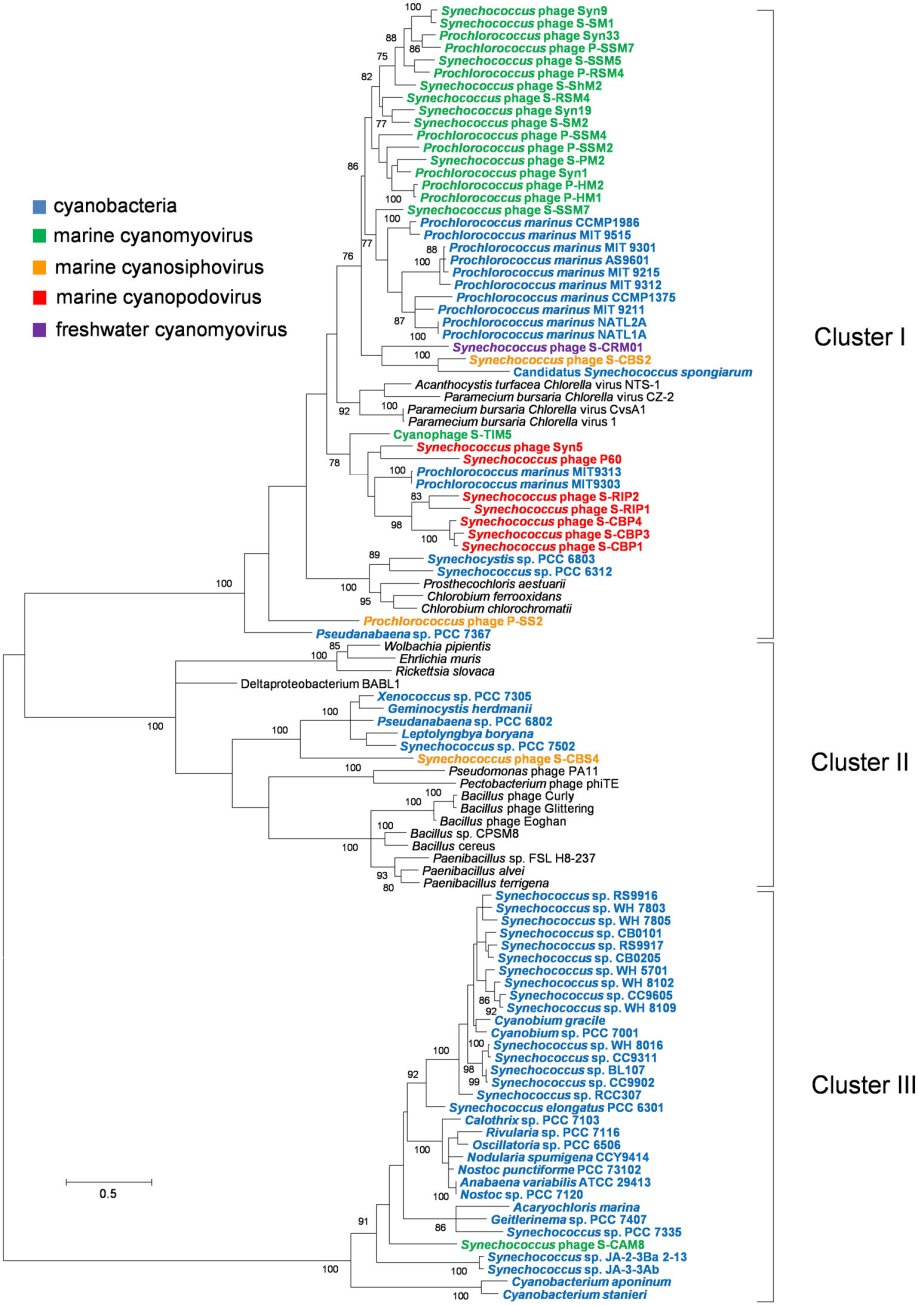
Maximum likelihood phylogenetic analysis of thymidylate synthase gene *thyX* in cyanophages and cyanobacteria. Cyanobacterial and cyanophage sequences were shown in color and other bacterial and viral sequences in black. Bootstrap test values higher than 75% were shown.

All the five *Synechococcus* podoviruses in cluster MPP-B and two in MPP-A encode a thymidylate synthase gene, *thyX*, which is located at the right end of each chromosome (Fig 1). Instead of encoding a *thyX*, all the *Prochlorococcus* podoviruses in cluster MPP-B have a *talC* at the same locus (Fig 1). It is likely that one of the two genes was replaced by another. The *thyX* genes are extremely divergent [57]. Thus, it is not surprised that the *thyX* sequences from cyanobacteria and cyanophages fell into three discrete clusters or versions (Fig 6). The sequences from most of cyanobacteria (cluster III) were grouped together and likely follow a vertical descent in evolution, while those from *Synechococcus* podoviruses and most marine cyanomyoviruses were clustered with *Prochlorococcus* (cluster I). Moreover, two distant subclusters were emerged among these cyanophages and *Prochlorococcus*, one comprising marine cyanomyoviruses and *Prochlorococcus*, and the other one comprising *Synechococcus* podoviruses, two low-light *Prochlorococcus* and one *Synechococcus* myovirus (Fig 6, cluster I). Together, these phylogenetic patterns strongly support the horizontal transfer of *thyX* between cyanophage and *Prochlorococcus* [53]. Furthermore, none of *talC* or *thyX* was found in T7-like heterotrophic bacteria phages [58] or freshwater T7-like cyanophages [42, 43]. *talC* is a typical bacteria gene and the cyanophage-encoded version is thought to be of bacteria origin [20, 56]. Thus, it is unlikely that cyanopodoviruses inherited *talC* and *thyX* from their T7-like phage ancestor but possibly acquired them elsewhere. ThyX is an alternative type of thymidylate synthase which synthesizes the essential DNA precursor, thymidylate (dTMP), from uridylate (dUMP) [57]. Interestingly, the product of cyanophage *talC* was found to be involved in redirection of host metabolism, which could increase deoxynucleotide biosynthesis [26]. Likely, the two functionally different genes at a same genome locus may lead to similar roles during phage replication. *Prochlorococcus* and *Synechococcus* podoviruses may employ different mechanisms to overcome the shortage of deoxynucleotide.

Cyanophage-encoded host-like genes can be expressed during the infection cycle and are thought to be beneficial to phage fitness [24–26]. The local phosphorus stress could affect the distribution of phosphorus metabolism related genes among cyanomyovirus isolates [48] and communities [46] from different oceans. We also observed different occurrence trends of AMGs between the two cyanopodovirus clusters, that is, MPP-B phages tend to obtain a few AMGs which are absent or only sporadically exist in MPP-A phages. MPP-A and MPP-B cyanopodoviruses likely have differentiation in ecological prevalence as revealed that MPP-B appears to be the dominant cluster in marine habitats [14, 27, 28]. Moreover, the relative abundances of subclusters within MPP-B are highly variable in different environments [55]. For instance, the Chesapeake Bay phages (MPP-B4) were found to be predominant in that estuary but quite rare in coastal and open ocean waters [14, 28, 55]. Such distribution preference of cyanopodovirus genotypes might be closely related to that of their hosts [55], reflecting adaptation to hosts as well as to environment. The cyanopodovirus genomes share a conserved core genome and both the phylogenies of core genes and the presence/absence pattern of non-core genes could distinguish the clusters and subclusters. It is likely that the majority of non-core genes co-evolved with the core, possibly both driven by adaptation to factors such as host and environment.

## Conclusions

Podoviruses which infect marine *Synechococcus* and *Prochlorococcus* share a highly conserved genomic structure, despite differences in host systems and origins of habitat (estuarine or oceanic waters). Core genes make up half of genome length of marine cyanopodoviruses. Our whole genome phylogenetic analyses confirmed the divergence of two discrete clusters of marine cyanopodoviruses, MPP-A and MPP-B. MPP-B phages encode several accessory genes (i.e. *psbA*, *talC* and *thyX*), which can potentially provide phages with selection advantage for inhabiting nutrient poor marine environments. Future studies are needed to explore the role of phage-encoded auxiliary metabolic genes in the ecological distribution of cyanobacterial podoviruses.

## Supporting Information

S1 File All the COGs identified based on the 20 cyanopodovirus genomes analyzed in this study. (XLSX)
